# Supplementary material for: Distinct evolutionary trajectories in the Escherichia coli pangenome occur within sequence types
Source: Microb Genom. 2022 Nov 23;8(11):mgen000903. doi: 10.1099/mgen.0.000903 (PMC9836092; doi:10.1099/mgen.0.000903)
Supplement: Supplementary material 1 [file mgen-8-903-s001.pdf]

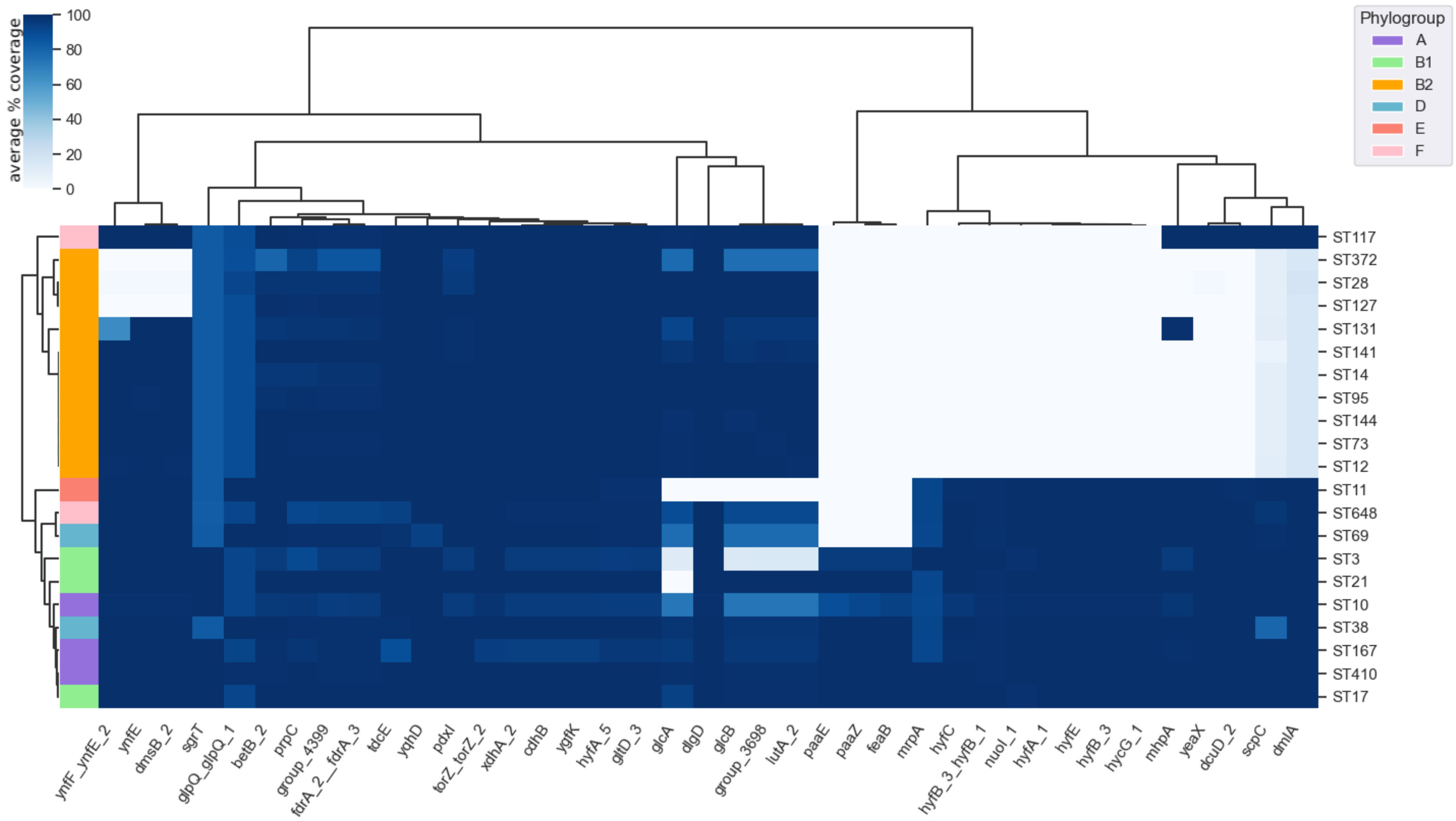

S 1. Hierarchically clustered heatmap of the average presence of energy production and conversion clusters (COG functional category C) from the ST410 core genome across 21 sequence types of *E. coli*.

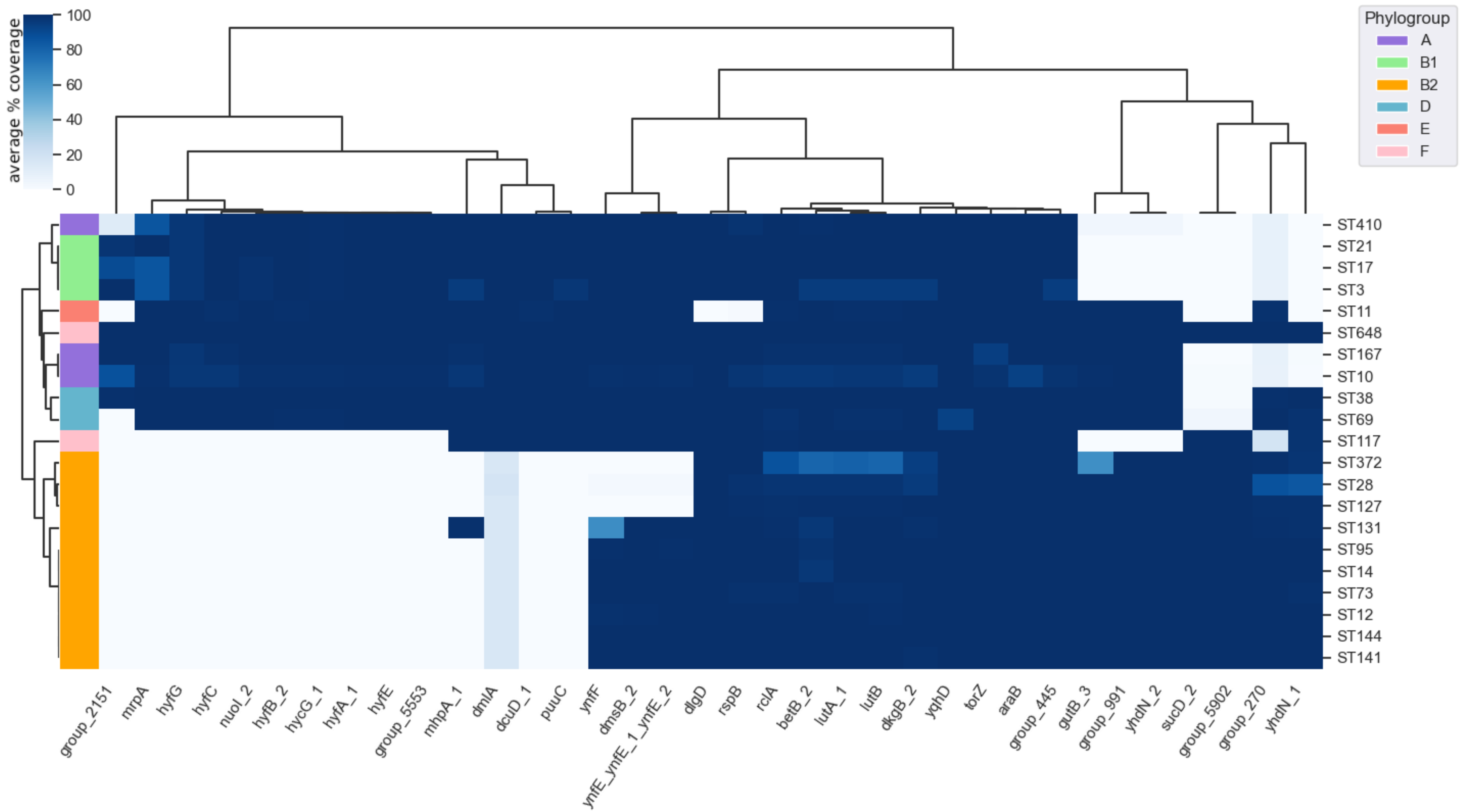

S 2. Hierarchically clustered heatmap of the average presence of energy production and conversion (COG functional category C) clusters from the ST648 core genome across 21 sequence types of *E. coli*.

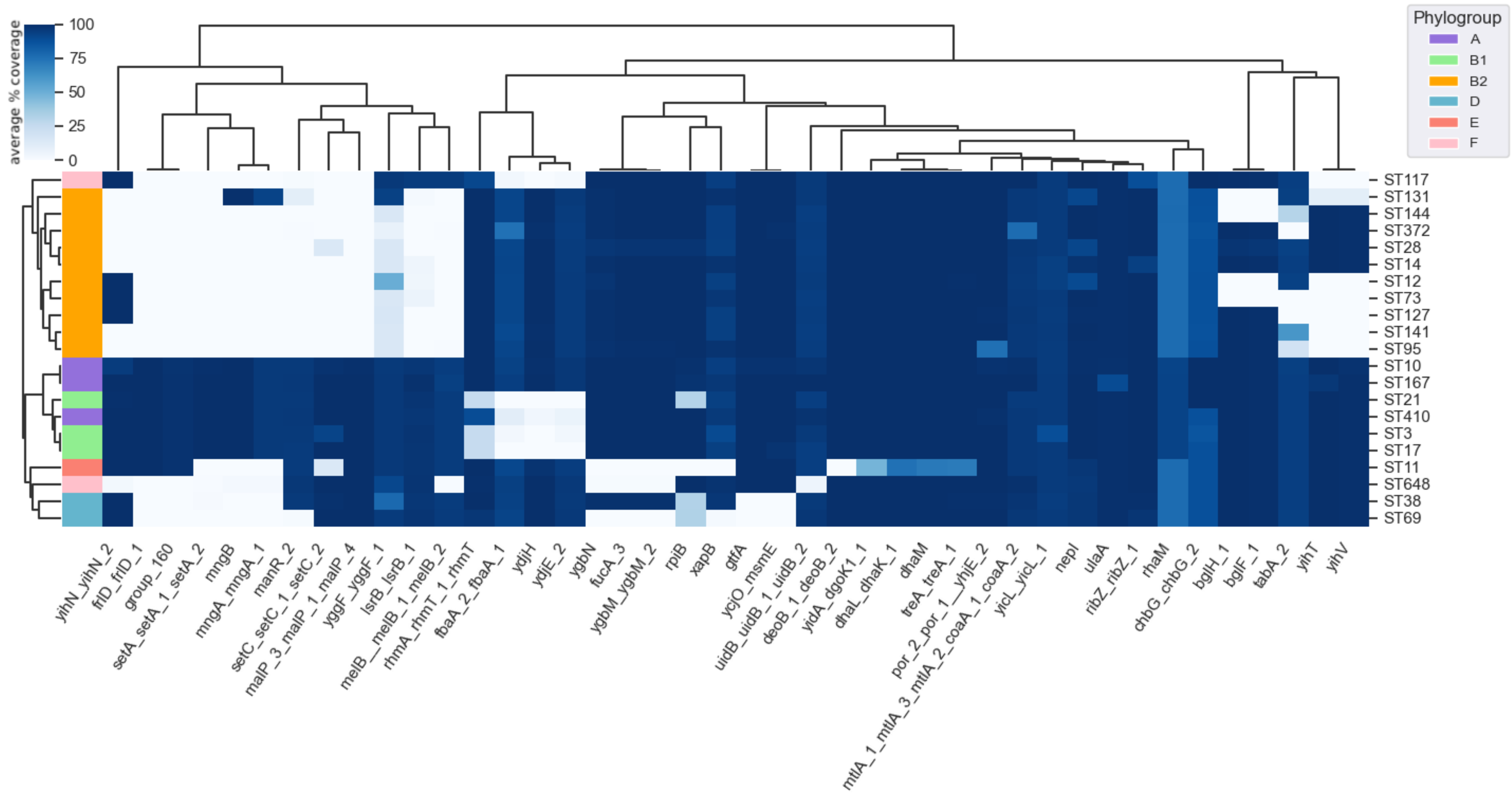

S 3. Hierarchically clustered heatmap of the average presence of carbohydrate transport and metabolism clusters (COG functional category G) from the ST10 core genome across 21 sequence types of *E. coli*.

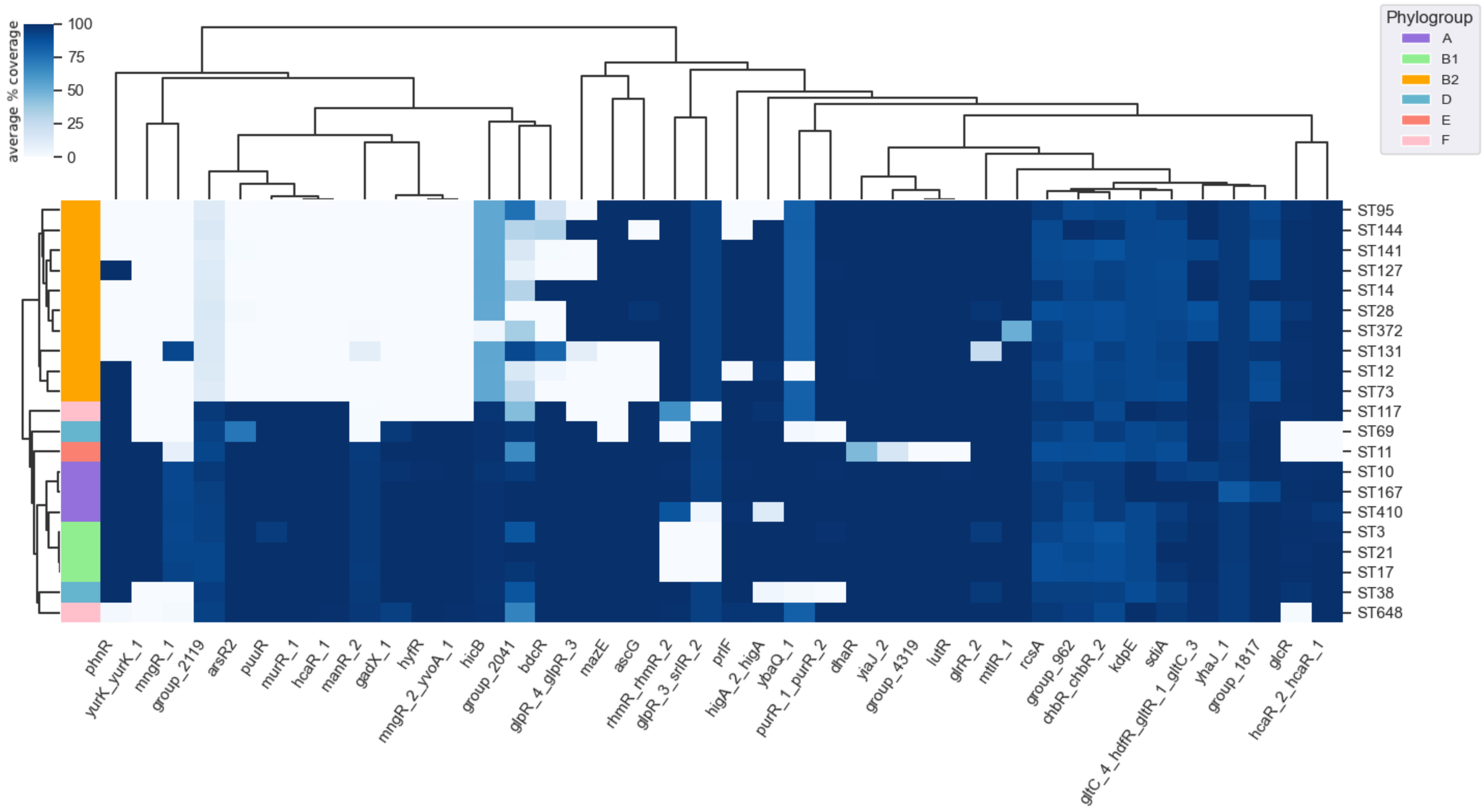

S 4. Hierarchically clustered heatmap of the average presence of transcription clusters (COG functional category K) from the ST10 core genome across 21 sequence types of *E. coli*.

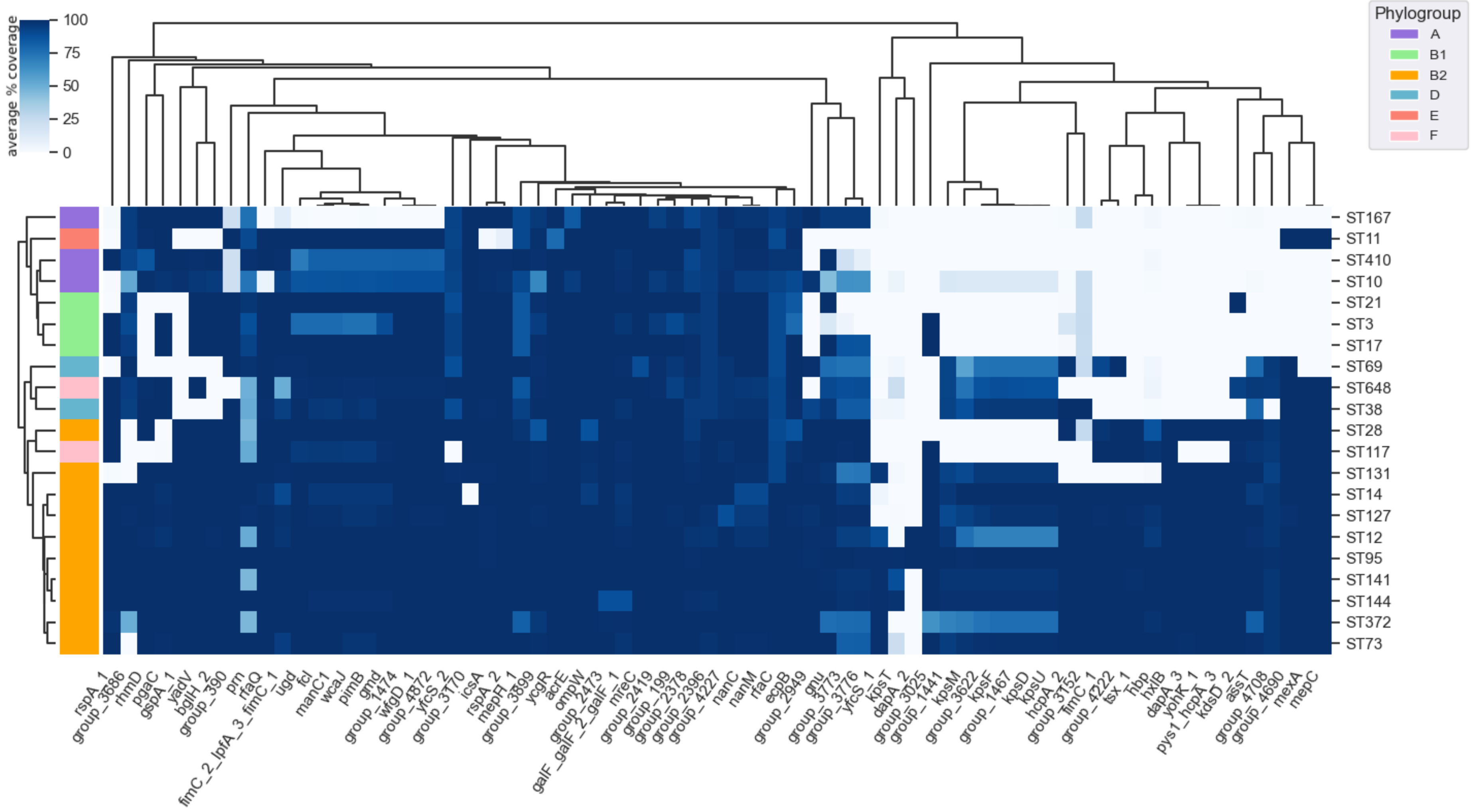

S 5. Hierarchically clustered heatmap of the average presence of cell wall/membrane/envelope biogenesis clusters (COG functional category M) from the ST95 core genome across 21 sequence types of *E. coli*.

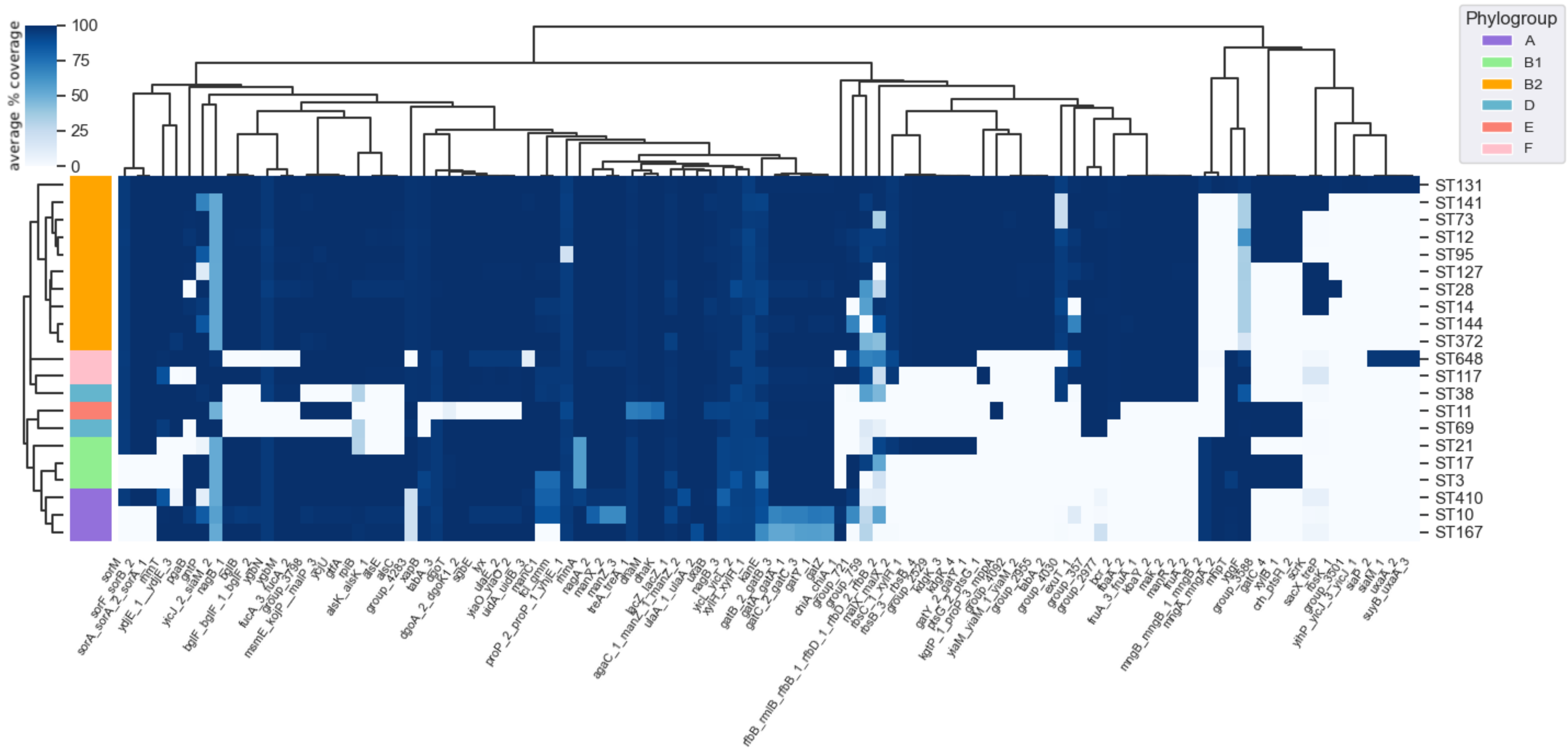

S 6. Hierarchically clustered heatmap of the average presence of carbohydrate transport and metabolism clusters (COG functional category G) from the ST131 core genome across 21 sequence types of *E. coli*.
